# 3-(2-Methyl-1,3-benzo­thia­zol-3-ium-3-yl)propane-1-sulfonate monohydrate

**DOI:** 10.1107/S1600536814011660

**Published:** 2014-05-24

**Authors:** Guo-Cui Zhang, Ming Kong, Sheng-Li Li

**Affiliations:** a Deparment of Chemistry, Anhui University, Hefei 230039, People’s Republic of China, Key Laboratory of Functional Inorganic Materials, Chemistry, Hefei 230039, People’s Republic of China

## Abstract

In the title hydrated zwitterion, C_11_H_13_NO_3_S_2_·H_2_O, the N—C—C—C and C—C—C—S torsion angles in the side-chain are 171.06 (14) and 173.73 (12)°, respectively. In the crystal, inversion-related mol­ecules are π-stacked with an inter­planar separation of 3.3847 (2) Å. O—H⋯O hydrogen bonds link inversion-related mol­ecules with a pair of water mol­ecules to form *R*_4_^2^(8) rings. The closest S⋯S contact is 3.4051 (15) Å between inversion-related mol­ecules.

## Related literature   

The crystal structure of a related benzo­thia­zole derivative is described by Lynch (2002 ▶). An analysis of bond angles in the thia­zole ring system has been given by Muir *et al.* (1987 ▶). Applications of benzo­thia­zole derivatives have been described by Vicini *et al.* (2003 ▶); Bondock *et al.* (2010 ▶); Paramashivappa *et al.* (2003 ▶) and Sayama *et al.* (2002 ▶). 
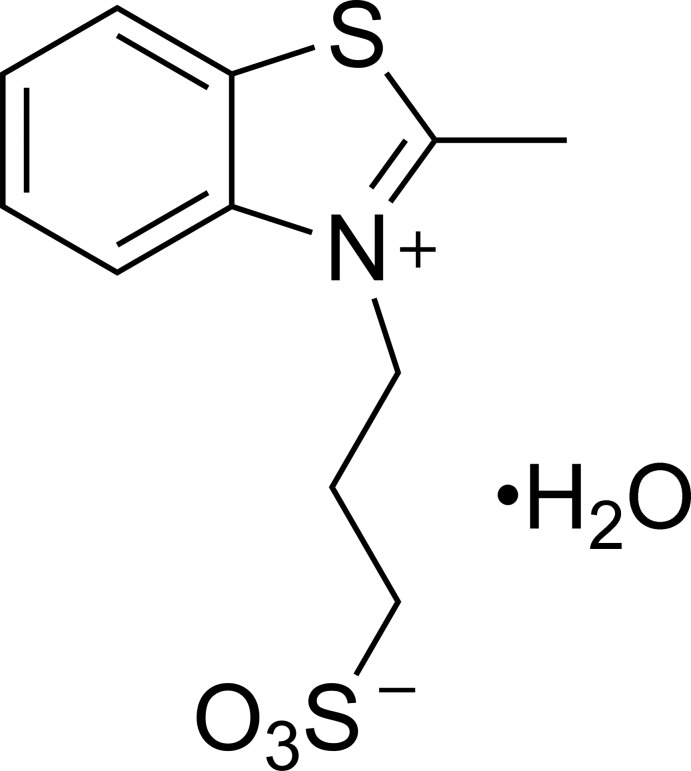


## Experimental   

### 

#### Crystal data  

C_11_H_13_NO_3_S_2_·H_2_O
*M* *_r_* = 289.36
Monoclinic, 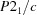

*a* = 10.936 (5) Å
*b* = 8.708 (5) Å
*c* = 13.794 (5) Å
β = 109.529 (5)°
*V* = 1238.0 (10) Å^3^
*Z* = 4
Mo *K*α radiation
μ = 0.44 mm^−1^
*T* = 296 K
0.30 × 0.20 × 0.20 mm

#### Data collection  

Bruker SMART CCD area-detector diffractometer
Absorption correction: multi-scan (*SADABS*; Bruker, 2001 ▶) *T*_min_ = 0.880, *T*_max_ = 0.919
8500 measured reflections
2182 independent reflections
2105 reflections with *I* > 2sσ(*I*)
*R*_int_ = 0.016

#### Refinement  

*R*[*F* ^2^ > 2σ(*F* ^2^)] = 0.030
*wR*(*F* ^2^) = 0.080
*S* = 1.01
2182 reflections
164 parameters
H-atom parameters constrained
Δρ_max_ = 0.31 e Å^−3^
Δρ_min_ = −0.42 e Å^−3^

### 

Data collection: *SMART* (Bruker, 2007 ▶); cell refinement: *SAINT* (Bruker, 2007 ▶); data reduction: *SAINT*; program(s) used to solve structure: *SHELXS97* (Sheldrick, 2008 ▶); program(s) used to refine structure: *SHELXL97* (Sheldrick, 2008 ▶); molecular graphics: *SHELXTL* (Sheldrick, 2008 ▶); software used to prepare material for publication: *SHELXTL*.

## Supplementary Material

Crystal structure: contains datablock(s) I, Global. DOI: 10.1107/S1600536814011660/pk2524sup1.cif

Structure factors: contains datablock(s) I. DOI: 10.1107/S1600536814011660/pk2524Isup2.hkl

Supporting information file. DOI: 10.1107/S1600536814011660/pk2524Isup3.cml

CCDC reference: 1004303

Additional supporting information:  crystallographic information; 3D view; checkCIF report

## Figures and Tables

**Table 1:** Hydrogen-bond geometry (Å, °)

*D*—H⋯*A*	*D*—H	H⋯*A*	*D*⋯*A*	*D*—H⋯*A*
O4—H11⋯O3^i^	0.76	2.07	2.831 (2)	176
O4—H12⋯O3	0.82	2.21	2.994 (3)	160
C3—H3*A*⋯O1^ii^	0.97	2.39	3.269 (3)	151
C4—H4*C*⋯O4^iii^	0.96	2.54	3.487 (3)	169

Symmetry codes: (i) 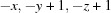; (ii) 
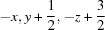
; (iii) 
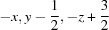
.

**Fig. 1.**
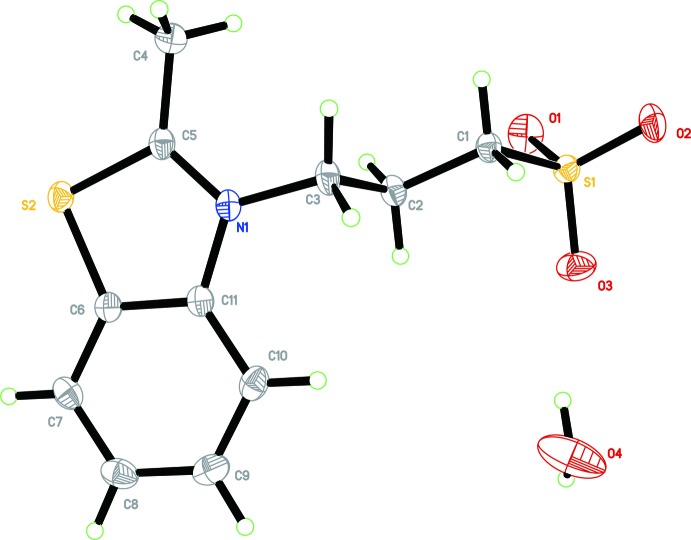
: The molecular structure of the title compound showing 30% probability displacement ellipsoids.

**Fig. 2.**
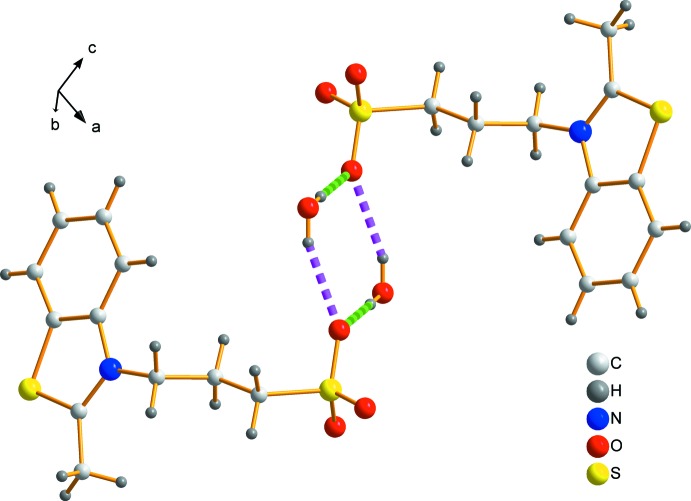
: View of the R24(8) ring formed by O4—H12···O3 and O4—H11···O3i intermolecular interactions, showing O—H···O hydrogen-bonding interactions as dashed lines.

**Fig. 3.**
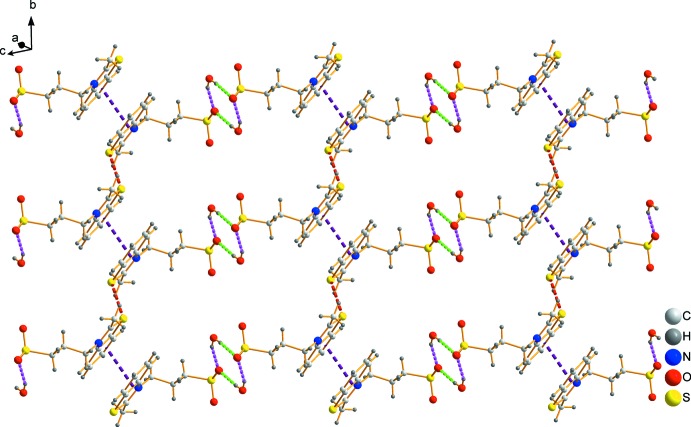
: Packing diagram of the title compound.

## supplementary crystallographic information

### 1. Comment

Benzothiazole, a small and simple heterocyclic molecule, has raised considerable interest. It can be used to synthesize some Schiff bases (Vicini *et al.*, 2003), and other derivatives that are antimicrobial (Bondock *et al.*, 2010) and bioactive (Paramashivappa *et al.*, 2003). They have also been used in dye-sensitized solar cells (Sayama *et al.*, 2002). Spurred by this, we synthesized 3-(2-methylbenzo[*d*]thiazol-3-ium-3-yl)propane-1-sulfonate (Fig. 1), which contains a sulfonic group, with the aim of increased solubility. The single-crystal structure contains one water molecule. Comparing with C_7_H_5_N_3_O_2_S_1_·H_2_O (Lynch, 2002), both of them are in a hydrogen-bonding network with water molecules. The water H atoms are connected with O atoms of sulfonic moieties and the molecules are interconnected, *via* hydrogen bonds (Table 1) [O4—H11···O3^i^, symmetry codes: (i) -*x*, -*y* + 1, -*z* + 1; C3—H3A···O1^ii^, symmetry codes: (ii) -*x*, *y* + 1/2, -*z* + 3/2; C4—H4C···O4^iii^, symmetry codes: (iii) -*x*, *y* - 1/2, -*z* + 3/2]. There is a *R*^2^_4_(8) ring formed by hydrogen-bonded water to O3—S1 interactions. Two characteristic O4—H12···O3 and O4—H11···O3^i^ distances are 2.994 (3) Å and 2.831 (2) Å, respectively (Fig.2). In the crystal, inversion related (1-x,1-y,2-z) molecules are π-stacked with an interplanar separation of 3.3847 (2) Å. O—H···O hydrogen bonds link inversion-related (-x,1-y,1-z) molecules with a pair of water molecules to form *R*^2^_4_(8) rings. The closest ring S···S contact is 3.4051 (15) Å between inversion-related (1-x,-y,2-z) molecules (Fig.3). The bond length between N1 and C5 [1.3216 (2) Å] indicates some double bond character and is conjugated with neighbouring bonds. The two distances of S2—C6 and S2—C5 are nearly the same [1.7327 (19) Å and 1.7024 (18) Å, respectively]. In addition, the large size of the S atom compared with N results in a reduction of the C5—S2—C6 angle [91.069 (8)°] compared with the C5—N1—C11 angle [114.001 (14)°] in thiazole ring. This reveals that the S atom might be using unhybridized p-orbitals for bonding (Muir *et al.*, 1987).

### 2. Experimental

The title complex, 3-(2-methylbenzo[*d*]thiazol-3-ium-3-yl)propane-1-sulfonate, was prepared by mixing 2-methylbenzo[*d*]thiazole (1.49 g, 0.010 mol) with 1,2-oxathiolane 2,2-dioxide (1.47 g, 0.012 mol) in toluene (20 ml). The mixture was heated to reflux for 4 h. After the reaction was complete, the solution was cooled to room temperature. The mixture was filtered and washed with ethanol 3 times to give a white solid. Colorless block-shaped crystals were grown by slow evaporation an acetonitrile/ethanol mixture. ^1^H NMR: (400 Hz, DMSO-d_6_), d(p.p.m.): 8.43 (t, 2H), 7.90 (t, 1H), 7.80 (t, 1H), 4.90 (t, 2H), 3.20 (s, 3H), 2.65 (t, 2H), 2.15 (q, 2H).

### 3. Refinement

The water H atoms were located in a difference map and refined isotropically with *U*_iso_(H) = 1.5 *U*_eq_(O). Other hydrogens were placed in geometrically idealized positions (C—H = 0.93–0.97 Å) and allowed to ride on their parent atoms with *U*_iso_(H) = 1.2 *U*_eq_(C) or 1.5*U*_eq_(C_Me_).

### Crystal data

**Table:**

C_11_H_13_NO_3_S_2_·H_2_O	*F*(000) = 608
*M**_r_* = 289.36	*D*_x_ = 1.552 Mg m^−^^3^
Monoclinic, *P*2_1_/*c*	Mo *K*α radiation, λ = 0.71069 Å
Hall symbol: -P 2ybc	Cell parameters from 7517 reflections
*a* = 10.936 (5) Å	θ = 2.8–27.1°
*b* = 8.708 (5) Å	µ = 0.44 mm^−^^1^
*c* = 13.794 (5) Å	*T* = 296 K
β = 109.529 (5)°	Block, white
*V* = 1238.0 (10) Å^3^	0.30 × 0.20 × 0.20 mm
*Z* = 4	

### Data collection

**Table:**

Bruker SMART CCD area-detector diffractometer	2182 independent reflections
Radiation source: fine-focus sealed tube	2105 reflections with *I* > 2sσ(*I*)
Graphite monochromator	*R*_int_ = 0.016
φ and ω scans	θ_max_ = 25.0°, θ_min_ = 2.0°
Absorption correction: multi-scan (*SADABS*; Bruker, 2001)	*h* = −13→12
*T*_min_ = 0.880, *T*_max_ = 0.919	*k* = −10→10
8500 measured reflections	*l* = −16→16

### Refinement

**Table:**

Refinement on *F*^2^	Primary atom site location: structure-invariant direct methods
Least-squares matrix: full	Secondary atom site location: difference Fourier map
*R*[*F*^2^ > 2σ(*F*^2^)] = 0.030	Hydrogen site location: inferred from neighbouring sites
*wR*(*F*^2^) = 0.080	H-atom parameters constrained
*S* = 1.01	*w* = 1/[σ^2^(*F*_o_^2^) + (0.0449*P*)^2^ + 0.799*P*] where *P* = (*F*_o_^2^ + 2*F*_c_^2^)/3
2182 reflections	(Δ/σ)_max_ < 0.001
164 parameters	Δρ_max_ = 0.31 e Å^−^^3^
0 restraints	Δρ_min_ = −0.42 e Å^−^^3^

### Special details

**Table:**

Geometry. All e.s.d.'s (except the e.s.d. in the dihedral angle between two l.s. planes) are estimated using the full covariance matrix. The cell e.s.d.'s are taken into account individually in the estimation of e.s.d.'s in distances, angles and torsion angles; correlations between e.s.d.'s in cell parameters are only used when they are defined by crystal symmetry. An approximate (isotropic) treatment of cell e.s.d.'s is used for estimating e.s.d.'s involving l.s. planes.
Refinement. Refinement of *F*^2^ against ALL reflections. The weighted *R*-factor *wR* and goodness of fit *S* are based on *F*^2^, conventional *R*-factors *R* are based on *F*, with *F* set to zero for negative *F*^2^. The threshold expression of *F*^2^ > σ(*F*^2^) is used only for calculating *R*-factors(gt) *etc*. and is not relevant to the choice of reflections for refinement. *R*-factors based on *F*^2^ are statistically about twice as large as those based on *F*, and *R*- factors based on ALL data will be even larger.

### Fractional atomic coordinates and isotropic or equivalent isotropic displacement parameters (Å^2^)

**Table:**

	*x*	*y*	*z*	*U*_iso_*/*U*_eq_	
C1	−0.04082 (15)	0.49554 (19)	0.80688 (12)	0.0271 (3)	
H1A	−0.0210	0.6044	0.8110	0.033*	
H1B	−0.0771	0.4720	0.8604	0.033*	
C2	0.08437 (15)	0.4060 (2)	0.82760 (12)	0.0294 (4)	
H2A	0.1180	0.4207	0.7715	0.035*	
H2B	0.0682	0.2972	0.8327	0.035*	
C3	0.18249 (16)	0.46262 (19)	0.92775 (13)	0.0301 (4)	
H3A	0.2076	0.5670	0.9186	0.036*	
H3B	0.1426	0.4636	0.9809	0.036*	
C4	0.23671 (18)	0.2245 (2)	1.09534 (14)	0.0388 (4)	
H4A	0.2531	0.2983	1.1499	0.058*	
H4B	0.2549	0.1232	1.1240	0.058*	
H4C	0.1474	0.2306	1.0522	0.058*	
C5	0.32123 (15)	0.25761 (19)	1.03338 (12)	0.0272 (3)	
C6	0.49490 (15)	0.26655 (18)	0.95809 (12)	0.0264 (3)	
C7	0.60073 (16)	0.2548 (2)	0.92365 (13)	0.0333 (4)	
H7	0.6661	0.1832	0.9517	0.040*	
C8	0.60501 (18)	0.3533 (2)	0.84639 (14)	0.0390 (4)	
H8	0.6733	0.3465	0.8207	0.047*	
C9	0.50797 (19)	0.4631 (2)	0.80639 (13)	0.0383 (4)	
H9	0.5145	0.5296	0.7556	0.046*	
C10	0.40270 (17)	0.4758 (2)	0.83992 (13)	0.0328 (4)	
H10	0.3387	0.5494	0.8131	0.039*	
C11	0.39668 (15)	0.37330 (18)	0.91581 (12)	0.0258 (3)	
N1	0.29980 (13)	0.36398 (15)	0.96126 (10)	0.0257 (3)	
O1	−0.18774 (14)	0.29348 (16)	0.68281 (11)	0.0490 (4)	
O2	−0.26970 (12)	0.55228 (16)	0.68129 (11)	0.0447 (3)	
O3	−0.10098 (14)	0.50116 (19)	0.60890 (10)	0.0487 (4)	
O4	0.09084 (19)	0.6948 (3)	0.55198 (17)	0.0951 (8)	
H11	0.0932	0.6459	0.5068	0.143*	
H12	0.0523	0.6424	0.5819	0.143*	
S1	−0.15982 (4)	0.45636 (5)	0.68522 (3)	0.02836 (14)	
S2	0.46338 (4)	0.16183 (5)	1.05392 (3)	0.02926 (14)	

### Atomic displacement parameters (Å^2^)

**Table:**

	*U*^11^	*U*^22^	*U*^33^	*U*^12^	*U*^13^	*U*^23^
C1	0.0260 (8)	0.0278 (8)	0.0252 (8)	0.0026 (7)	0.0053 (6)	−0.0020 (6)
C2	0.0262 (8)	0.0304 (8)	0.0289 (8)	0.0044 (7)	0.0058 (7)	−0.0041 (7)
C3	0.0282 (8)	0.0283 (8)	0.0295 (8)	0.0087 (7)	0.0039 (7)	−0.0034 (6)
C4	0.0365 (9)	0.0464 (11)	0.0353 (9)	0.0047 (8)	0.0144 (8)	0.0022 (8)
C5	0.0269 (8)	0.0271 (8)	0.0238 (7)	0.0020 (6)	0.0035 (6)	−0.0043 (6)
C6	0.0265 (8)	0.0243 (8)	0.0254 (8)	−0.0021 (6)	0.0049 (6)	−0.0043 (6)
C7	0.0266 (8)	0.0352 (9)	0.0368 (9)	−0.0014 (7)	0.0089 (7)	−0.0075 (7)
C8	0.0353 (10)	0.0461 (11)	0.0382 (10)	−0.0124 (8)	0.0156 (8)	−0.0107 (8)
C9	0.0457 (11)	0.0394 (10)	0.0283 (9)	−0.0146 (8)	0.0102 (8)	−0.0020 (7)
C10	0.0351 (9)	0.0292 (9)	0.0269 (8)	−0.0042 (7)	0.0010 (7)	0.0001 (7)
C11	0.0253 (8)	0.0245 (8)	0.0244 (7)	−0.0025 (6)	0.0039 (6)	−0.0052 (6)
N1	0.0247 (7)	0.0250 (7)	0.0243 (6)	0.0036 (5)	0.0038 (5)	−0.0029 (5)
O1	0.0500 (8)	0.0333 (7)	0.0562 (9)	−0.0105 (6)	0.0079 (7)	−0.0086 (6)
O2	0.0295 (7)	0.0515 (8)	0.0460 (8)	0.0109 (6)	0.0032 (6)	0.0044 (6)
O3	0.0504 (8)	0.0678 (10)	0.0303 (7)	−0.0090 (7)	0.0166 (6)	0.0000 (6)
O4	0.0798 (13)	0.1204 (18)	0.1083 (16)	−0.0428 (13)	0.0622 (12)	−0.0642 (14)
S1	0.0254 (2)	0.0311 (2)	0.0257 (2)	−0.00197 (15)	0.00465 (17)	−0.00040 (15)
S2	0.0295 (2)	0.0272 (2)	0.0296 (2)	0.00697 (16)	0.00785 (17)	0.00262 (16)

### Geometric parameters (Å, º)

**Table:**

C1—C2	1.518 (2)	C6—C7	1.394 (2)
C1—S1	1.7793 (16)	C6—S2	1.7329 (17)
C1—H1A	0.9700	C7—C8	1.381 (3)
C1—H1B	0.9700	C7—H7	0.9300
C2—C3	1.521 (2)	C8—C9	1.397 (3)
C2—H2A	0.9700	C8—H8	0.9300
C2—H2B	0.9700	C9—C10	1.381 (3)
C3—N1	1.484 (2)	C9—H9	0.9300
C3—H3A	0.9700	C10—C11	1.394 (2)
C3—H3B	0.9700	C10—H10	0.9300
C4—C5	1.482 (2)	C11—N1	1.402 (2)
C4—H4A	0.9600	O1—S1	1.4489 (16)
C4—H4B	0.9600	O2—S1	1.4495 (14)
C4—H4C	0.9600	O3—S1	1.4587 (14)
C5—N1	1.322 (2)	O4—H11	0.7630
C5—S2	1.7024 (17)	O4—H12	0.8203
C6—C11	1.393 (2)		
			
C2—C1—S1	114.11 (11)	C11—C6—S2	110.39 (12)
C2—C1—H1A	108.7	C7—C6—S2	128.39 (13)
S1—C1—H1A	108.7	C8—C7—C6	117.63 (17)
C2—C1—H1B	108.7	C8—C7—H7	121.2
S1—C1—H1B	108.7	C6—C7—H7	121.2
H1A—C1—H1B	107.6	C7—C8—C9	120.82 (17)
C1—C2—C3	108.81 (13)	C7—C8—H8	119.6
C1—C2—H2A	109.9	C9—C8—H8	119.6
C3—C2—H2A	109.9	C10—C9—C8	122.04 (17)
C1—C2—H2B	109.9	C10—C9—H9	119.0
C3—C2—H2B	109.9	C8—C9—H9	119.0
H2A—C2—H2B	108.3	C9—C10—C11	117.02 (16)
N1—C3—C2	111.67 (13)	C9—C10—H10	121.5
N1—C3—H3A	109.3	C11—C10—H10	121.5
C2—C3—H3A	109.3	C6—C11—C10	121.23 (16)
N1—C3—H3B	109.3	C6—C11—N1	111.50 (14)
C2—C3—H3B	109.3	C10—C11—N1	127.25 (15)
H3A—C3—H3B	107.9	C5—N1—C11	114.00 (13)
C5—C4—H4A	109.5	C5—N1—C3	123.89 (14)
C5—C4—H4B	109.5	C11—N1—C3	122.08 (13)
H4A—C4—H4B	109.5	H11—O4—H12	105.3
C5—C4—H4C	109.5	O1—S1—O2	113.43 (9)
H4A—C4—H4C	109.5	O1—S1—O3	112.69 (9)
H4B—C4—H4C	109.5	O2—S1—O3	112.28 (9)
N1—C5—C4	125.63 (15)	O1—S1—C1	106.94 (8)
N1—C5—S2	113.01 (12)	O2—S1—C1	105.11 (8)
C4—C5—S2	121.31 (13)	O3—S1—C1	105.63 (9)
C11—C6—C7	121.22 (16)	C5—S2—C6	91.07 (8)
			
S1—C1—C2—C3	173.73 (12)	C4—C5—N1—C3	−3.1 (2)
C1—C2—C3—N1	171.06 (14)	S2—C5—N1—C3	179.33 (11)
C11—C6—C7—C8	0.3 (2)	C6—C11—N1—C5	−0.11 (19)
S2—C6—C7—C8	−179.16 (13)	C10—C11—N1—C5	−178.36 (15)
C6—C7—C8—C9	1.6 (3)	C6—C11—N1—C3	−178.26 (13)
C7—C8—C9—C10	−1.6 (3)	C10—C11—N1—C3	3.5 (2)
C8—C9—C10—C11	−0.2 (2)	C2—C3—N1—C5	−100.77 (18)
C7—C6—C11—C10	−2.2 (2)	C2—C3—N1—C11	77.20 (19)
S2—C6—C11—C10	177.37 (12)	C2—C1—S1—O1	59.13 (15)
C7—C6—C11—N1	179.47 (14)	C2—C1—S1—O2	179.98 (13)
S2—C6—C11—N1	−1.01 (16)	C2—C1—S1—O3	−61.12 (15)
C9—C10—C11—C6	2.1 (2)	N1—C5—S2—C6	−1.51 (12)
C9—C10—C11—N1	−179.81 (15)	C4—C5—S2—C6	−179.18 (14)
C4—C5—N1—C11	178.76 (15)	C11—C6—S2—C5	1.41 (12)
S2—C5—N1—C11	1.21 (17)	C7—C6—S2—C5	−179.11 (16)

### Hydrogen-bond geometry (Å, º)

**Table:**

*D*—H···*A*	*D*—H	H···*A*	*D*···*A*	*D*—H···*A*
O4—H11···O3^i^	0.76	2.07	2.831 (2)	176
O4—H12···O3	0.82	2.21	2.994 (3)	160
C3—H3*A*···O1^ii^	0.97	2.39	3.269 (3)	151
C4—H4*C*···O4^iii^	0.96	2.54	3.487 (3)	169

Symmetry codes: (i) −*x*, −*y*+1, −*z*+1; (ii) −*x*, *y*+1/2, −*z*+3/2; (iii) −*x*, *y*−1/2, −*z*+3/2.
